# Dynamic Changes of Soil Surface Organic Carbon under Different Mulching Practices in Citrus Orchards on Sloping Land

**DOI:** 10.1371/journal.pone.0168384

**Published:** 2016-12-28

**Authors:** Chiming Gu, Yi Liu, Ibrahim Mohamed, Runhua Zhang, Xiao Wang, Xinxin Nie, Min Jiang, Margot Brooks, Fang Chen, Zhiguo Li

**Affiliations:** 1 Laboratory of Aquatic Botany and Watershed Ecology, Wuhan Botanical Garden, Chinese Academy of Sciences China, Wuhan, China; 2 Graduate School, University of Chinese Academy of Science, Beijing, China; 3 Oil Crops Research Institute of the Chinese Academy of Agricultural Sciences/Key Laboratory of Biology and Genetic Improvement of Oil Crops, Ministry of Agriculture, Wuhan, China; 4 Institute of Vegetable, Wuhan Academy of Agricultural Science and Technology, Wuhan, China, China; 5 Department of Biochemistry and Microbiology, Rhodes University, Grahamstown, South Africa; 6 China Program of International Plant Nutrition Institute, Wuhan, China; Tennessee State University, UNITED STATES

## Abstract

Mulching management has been used in many places all over the world to improve agricultural sustainability. However, the cycling of carbon in the soil under applications of mulch on sloping arable land is not yet fully understood. A four-year field experiment was carried out in Xiaofuling watershed of Danjiangkou reservoir in China. The object was to evaluate the effects of the application of straw mulch (ST) and grass mulch (GT) on dynamic changes in soil organic carbon and its fractions. Results showed that mulch applied on the soil surface increased the contents of SOC and its active fractions in the soil. Compared to the control without cover (CK), ST and GT treatments increased the contents of SOC, LOC, DOC, POC and EOC by 14.73%, 16.5%, 22.5%, 41.5% and 21%, respectively, in the 0–40 cm soil layer, and by 17%, 14%, 19%, and 30%, respectively, in the 0–100 cm soil layer. The contents of organic carbon and its active fractions decreased with increasing soil depth in all of the treatments. SOC was accumulated in the period of December to the following March. The contents of soil DOC and LOC were high in January to March, while the contents of soil POC and EOC were high in June to September. The relative contents of soil organic carbon fractions were POC > EOC > LOC > DOC over the four years. Straw mulching had no significant effect on the changes in soil organic carbon active fractions during the different periods. Based on this long-term field experiment in Danjiangkou reservoir, we found that straw mulching had a significant effect on soil, increasing SOC content and stock in slopping arable land, and that live grass mulching was more effective than rice straw mulching. We discuss possible optimal periods for the implementation of mulching practices on sloping land.

## Introduction

Soil erosion in hill and mountain regions is a major issue, especially when combined with climate change. It poses threats not only to sustainable agriculture but also to the functioning of hill ecosystems [1]. In respect of global carbon cycling, the dynamics of SOC resulting from soil erosion processes on sloping arable land is of particular interest [2–3], especially in cases where agricultural activity has disturbed natural systems. SOC has an important role in the restoration of degraded lands with soil erosion [4–7]. However, it also contributes to greenhouse gas CO_2_ emission in soil, which has a large impact on the climate of the earth [8].

Soil erosion on sloping land can be reduced and SOC content improved by selecting appropriate soil management methods and land cover patterns [9]. Mulching practices have been introduced and proven to be effective in controlling soil erosion, reducing soil moisture evapotranspiration, surface runoff, and increasing the infiltration of water [10]. However, currently there are conflicting opinions regarding the effect of mulching on SOC concentrations and/or stocks due to contradictory results: enhancement [11], suppression [12], or no effect [13]. This uncertainty may be related to the complex processes of soil C sequestration [14], especially unclear for the dynamics of soil labile C fractions such as light organic carbon (LOC), dissolved organic carbon (DOC), particulate organic carbon (POC) and easily oxidizable organic carbon (EOC). Previous studies indicated that these labile C fractions accounted for a small part of soil total organic carbon. However, they had high turnover rates and fast mobility, and were easily altered according to local climate, soil texture and soil management, especially in disturbed agricultural systems, including arable lands [15]. Thus they have a large effect on soil biochemical processes, nutrient and carbon cycling [16]. Soil carbon fractions respond more quickly to environmental changes than total organic carbon, and therefore play an important role in dynamic change in soil condition. Understanding the character of SOC and labile C fractions, and their relationships with soil properties, is vital for a better assessment of the effects of management on soil properties, nutrient cycles, soil erosion, and C sequestration of sloping arable land.

Danjiangkou reservoir is located in northwestern Hubei province, China, where more than 80% of the population inhabits hill and mountainous areas, which comprise more than 92% of the land area. Recent rapid economic expansion and a growing demand for food have led to an increase in utilization and development of sloping land in rural areas. Cultivation using traditional tillage practices as well as excessive use of fertilizer is common. Crop residue is often removed, either physically, or by burning in situ in the field. Soil erosion and the associated impact on the environment are made more severe by a lack of soil conservation practices. According to statistics [17], by 2013 soil erosion in Danjiangkou reservoir expanded to 39 thousand km^2^, which is 45% of the whole reservoir area. Loss of topsoil each year is up to 169 million tons, resulting in overall reduction in soil quality. Research regarding the use of mulch in agriculture has focused mainly on the physical properties of soil [10], soil erosion [12], environmental protection [12, 17] and soil fertility [13]. The processes of dynamic change in the organic carbon fractions of soil, particularly in the Danjiangkou reservoir area, have seldom been considered.

This field study was established in 2009 at a citrus orchard situated in the Xiaofuling watershed of the Danjiangkou reservoir area in Hubei province, China. Citrus is the primary economic crop in the region, Danjiangkou city being one of the main citrus producing areas of China. The area currently planted with citrus is approximately 20,000 hm^2^. The annual harvest is about 300,000 t and the total value of the crop is almost 200 million RMB [18]. The purpose of this study was to evaluate the effect of soil surface mulching practices on changes in soil SOC and its labile fractions on sloping arable lands. The specific objectives were to determine the short term effects of mulching application (crop residue mulching and grass mulching) in sloping land on: (1) temporal variation and distribution of soil profile of SOC, and C stocks; (2) dynamic changes of labile fractions of SOC including LOC, DOC, POC and EOC; (3) the relationships between different SOC concentrations and selected soil properties such as soil temperature, soil pH and soil water content.

## Materials and Methods

### Site description

**Fig 1:**
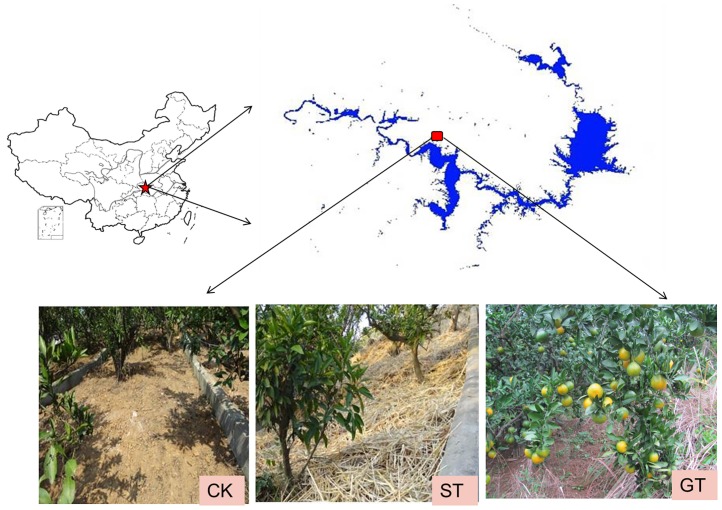
Location of sampling sites.

This study was conducted in 2012 in the Xiaofuling watershed of the Danjiangkou Reservoir area (32°36″–33°48″N, 110°59″–111°49″E) located in Xiaofuling county, Xijiadian town, Danjiangkou city, in Hubei province of central China (Fig 1). The experimental site is owned by Wuhan Botanical Garden, Chinese Academy of Sciences, China. The field studies did not involve endangered or protected species and no specific permits were required for the described field studies. The research area is characterized by a typical subtropical monsoon climate with a mean air temperature of 2.4°C in January and 28.4°C in July. Our experimental site was located about 300m above sea level, with an average slope of 15°. The soil was described by the Chinese soil classification system [19] as yellow cinnamon soil. It was made up of clay (14%), silt (23%) and sand (63%). The soil texture was classified as sandy loam soil, according to the soil texture classification standard of USA. Soil had a pH of 6.5 and a bulk density of 1.45 g cm^−3^. The amounts of SOM, total nitrogen, available phosphorus, and available potassium were 9.1 g kg^−1^, 0.9 g kg^−1^, 15.8 mg kg^−1^ and 110 mg kg^−1^, respectively. Mean annual precipitation for the last 10 years was approximately 813 mm, 84% of which fell between April and October. The equivalent value for 2012 was 687 mm, which was lower than the 10 year average.

### Experimental design

The experiment was designed as a randomized complete block with three treatments, in triplicate. The three treatments included: control treatment without any cover (CK), wheat straw mulching treatment (ST) and live grass clover mulching treatment (GT). Comparable amounts of dry biomass for ST and GT were determined in a preliminary experiment. Straw cover was applied at a rate of 375 g m^–2^ dry biomass for ST. Clover was plot-sown for GT, with a resulting dry biomass of approximately 360 g m^–2^, which was almost equal to the amounts of dry biomass applied in ST. The study site was an orchard with 10 year-old citrus trees of the local ‘Weizhang’ variety, which is widely grown in the region. Each experimental plot had an area of 40 m^2^ (4 m × 10 m), established in land with the same average slope of 15° and separated by concrete borders. Each plot contained 10 citrus trees planted in 2 rows of 5 trees. The space between rows was 2m and between plants 1.5m. The fertilizers urea (0.8 kg N/plant), superphosphate (0.2 kg P_2_O_5_/plant) and potassium chloride (0.2 kg K_2_O/plant) were added annually during the blooming stage in each treatment, by spot application. Any other management was identical in each treatment. Branches of the citrus trees were removed from the plots after trimming.

### Soil sampling and analysis

The soil was sampled to a depth of 100 cm at 5 randomly established locations in each plot. The samples were taken from below the mulch layer in those plots to which mulch had been applied. Samples were divided into subsamples for each of five depths: 0–20, 20–40, 40–60, 60–80and 80–100 cm. Sampling was repeated 7 times from January to December in 2012. A total of 315 samples were collected. After removing animal and plant residue and pebbles, soil samples were air-dried and put through a 100 or 200 mesh sieve for further analysis. For total biomass assessments in GT treatments, three 0.5 m^2^ areas located randomly at the top, middle and bottom of the slope were chosen in each plot. For aboveground biomass determination, the chosen areas were mowed. Underground biomass was collected by the soil drill method: the earth was drilled in the soil profile 0–50 cm and each 10 cm sampled as a layer. All clover roots were picked by hand from each sampling zone, placed in a numbered plastic bag and taken back to the laboratory. Leaves and roots were washed, dried and weighed, and recorded as aboveground and underground biomass respectively. Total biomass for each plot was calculated as the sum of both aboveground and underground biomass.

LOC was determined as described by Janzen et al. [20]. Representative soil samples (10 g, < 2 mm) were dispersed in 40 ml NaI solution with a specific gravity of 1.7 g cm^–3^. Suspensions were allowed to oscillate for 1 hour at a speed of 250 r min^–1^ before centrifuging for 10 min at 15000 r min^–1^. Suspended material (light fraction) was removed by suction and this process was repeated 3 times on the heavy fraction (unsuspended material). The light fraction material was washed, dried, ground and analyzed for total C using a total organic carbon analyzer.

DOC was extracted from 10 g of air-dried soil with distilled water using a ratio of 1:5 (soil: water) at 25°C [21]. After shaking for half an hour at a speed of 250 r min^–1^ and centrifuging for 10 min at 15000 r min^–1^, the suspended solution was filtered using a 0.45 μm membrane and analyzed for total C using a total organic carbon analyzer.

EOC was measured as described by Blair et al. [22]. Finely ground air-dried soil samples were reacted with 333 mmol L^–1^ KMnO_4_ by shaking at 60 r min^–1^ for 1 h. The suspension was then centrifuged at 2000 r min^–1^ for 10 min, diluted, and optical density measured at wavelength 565 nm using a spectrophotometer.

POC was measured using 20 g of dry soil dispersed in 60 ml of sodium hexametaphosphate (5 g L^–1^) shaking on a reciprocating shaker (90 r min^–1^) for 18 h [23]. The soil suspension was poured over a 100 μm screen using a flow of distilled water to ensure separation. All material remaining on the screen was washed into a dry plate, oven dried at 50°C for 48 h and ground, to determine C content.

**Fig 2:**
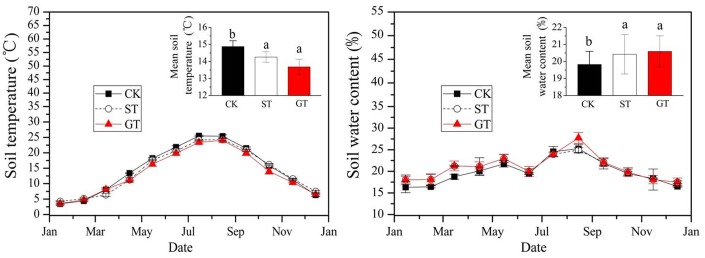
Monthly mean soil temperatures and soil water content in 2012.

SOC content was determined according to the K_2_Cr_2_O_7_–H_2_SO_4_ wet oxidation method of Walkley and Black [24]. Soil temperature was determined using a Li-6400 temperature probe and soil water content was determined gravimetrically by oven-drying (105°C for 24 h). The soil pH (1:5) and bulk density (BD) were measured. Total nitrogen (TN) concentration of the samples was determined by the Kjeldahl method after extraction by heating digestion with H_2_SO_4_-H_2_O_2_. Available phosphorus (AP) concentration of the samples was determined by the Olsen method after extraction with 0.03 mol L^–1^ NH_4_F 0.025 mol L^–1^ HCL, and available potassium (AK) concentration of the samples was determined by flame photometry after extraction with 1 mol L^–1^ NH_4_OAc [25]. Air temperature, and soil temperature at a depth of 5 cm were recorded at 0.5 h intervals by the StowAway TidbiT temperature recorder., The values recorded during the 24 h period were averaged to calculate mean daily temperature, shown in Fig 2.

### Statistical analysis

Means for the treatments were compared using the Least Significant Differences (LSD) and significant differences were determined at P < 0.05 probability level. All data was analyzed using the SPSS 16.0 statistical package (SPSS Inc., Chicago, IL, US) and Microsoft Excel 2010.

## Results and Discussions

### Soil properties

Treatments with ST and GT significantly affected soil properties in the surface 0 – 20cm layer (Table 1).

**Table 1:** Soil properties in surface layer (0–20cm) of the sampling sites.

Treatment	BD (g cm^–3^)	pH	TC (g kg^–1^)	TN (g kg^–1^)	C/N	Available P (mg kg^–1^)	Available K (mg kg^–1^)
CK	1.45 a*	6.5 a	6.70 c	0.88 b	7.61 a	16.0 b	106.3 c
ST	1.36 b	6.4 a	7.55 b	1.21 a	6.24 b	17.2 a	148.5 a
GT	1.41 a	5.9 b	7.91 a	1.17 a	6.76 b	14.2 c	125.2 b

*different letters indicate significant differences at p < 0.05 (same hereinafter)

In comparison with CK, the use of ST and GT both lowered soil BD values, but in the case of ST there was a significant difference. This agreed with the results of Unger and Jones [26], where mulching treatments with residue or straw decreased soil BD. The decrease of BD under straw mulching treatment is likely to be due to mulch lowering the kinetic energy of rain. Heavy rain could cause low levels of soil compaction, and decomposed straw residue provides a loose protective layer above the soil. Decrease in pH of the surface layer in the GT treatment might be attributed to the H^+^ or acid exudates released by clover roots. Jaillard et al. reported similar results [27].

ST and GT treatments increased soil TC and TN, but decreased the C/N ratio, in comparison to CK. This indicated that both ST and GT caused a greater increase in soil TN than soil TC. The increase in TC and TN under ST and GT compared to the control is mainly due to the organic matter derived from straw and biomass of clover [28], and to N fixation both above and below the ground. Soil available P and K was significantly higher in ST treatment than that in CK and GT which may result from P and K nutrient release due to straw decomposition. Larson et al. [29] reported a similar increase in soil P and K contents under straw mulch. In the GT treatment P and K may have been taken up by clover. This would explain why the P content in soil in the GT treatment was lower than that in other treatments. K is easily released back into the soil [30], hence the available K in the GT treatment was not as low as the P.

### Soil organic carbon concentration

**Fig 3:**
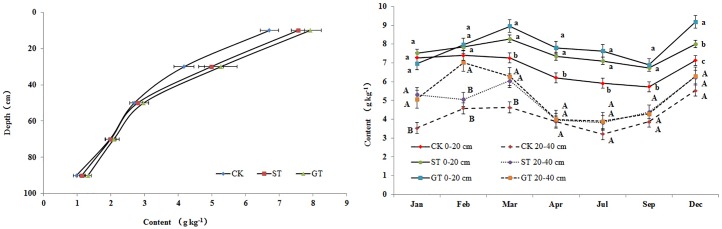
Changes of soil total organic carbon. Note: For each month, different uppercase letters represent significant differences in content in 20–40 cm soil layer of different treatments; different lower case letters represent significant differences in content in 0–20 cm soil layer of different treatments (hereinafter).

Distribution and seasonal dynamics of SOC in different mulching treatments are shown in Fig 3. SOC concentration in all treatments decreased with soil depth. The significant differences of SOC among treatments were solely at depths of 0–40 cm, where soil physicochemical properties (Table 1) changed. Further changes would have occurred following activity by microorganisms. Average SOC content at depths of 0–40cm in ST and GT were 6.26 g kg^–1^ and 6.59 g kg^–1^ respectively, significantly higher than that of 5.44 g kg^–1^ in CK (Fig 3). The use of ST and GT increased SOC by 15.15% and 21.14% respectively. In the course of the growing season, SOC concentrations in all treatments presented substantial changes with seasons. The maximum SOC was recorded in the dry and cold season, and the minimum in the warm and wet season. Similar results were reported by Bastian [31]. Mulching treatments did not alter the seasonal trends of SOC. These results indicated that the determining factors of SOC seasonal dynamics were probably soil moisture and soil temperature, and that mulching treatments merely served to increase the rate of accumulation of SOC at each time due to greater input of organic material and reduction of SOC decomposition, when compared to CK.

When comparing mulching treatments, SOC of the 0–40 cm soil layer in the GT treatment was significantly higher than that in the ST treatment, indicating that GT had a greater potential to increase SOC content in surface soil than ST. Similar results showing greater increase of SOC in GT than in ST have been observed in other studies, e.g., in the semiarid Loess Plateau of China [32] and in the southeast of England [33]. In the current study, the greater accumulation of SOC in topsoil under GT is likely to have resulted from the slower decomposition rate of SOC compared with the ST treatments, due to lower soil temperatures in GT than in ST (Fig 2). The differences in soil temperature between the two mulching treatments were sustained throughout the year. Another possible explanation for SOC being higher in GT is that although the dry weight of biomass (including root biomass in the GT) applied was almost the same during the whole growth season for both GT and ST (375 g m^–2^ and 360 g m^–2^ respectively), the eventual sum of organic material available on the soil in ST was likely to have been lower. Straw residues were loose on the soil surface. Small debris and particles of organic matter resulting from decomposition were easily lost with surface runoff, exacerbated in the rainy season by the slope of the land [34]. In contrast, adoption of GT resulted in aboveground organic materials being anchored by roots in the surface soil. Organic matter from roots and their secretions in the subsoil layer was stored in the soil, and thus contributed to a decrease in the losses associated with runoff.

### Changes in soil labile organic carbon

**Fig 4:**
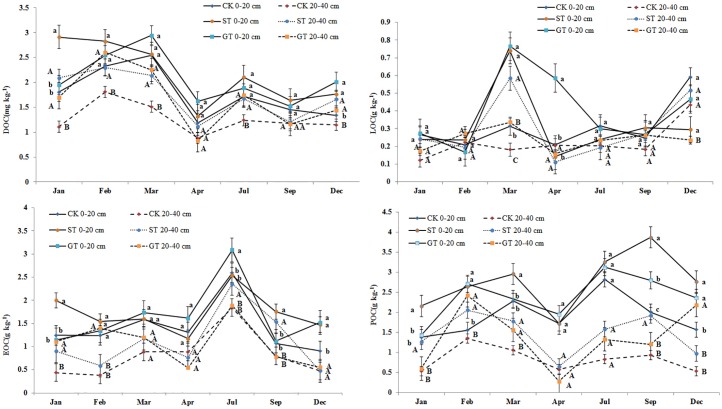
Dynamic changes of carbon fractions.

Various studies have shown that mulching application could significantly affect the concentration of the labile SOC fractions, especially in the top layers of soil [35–37]. The data of C fractions under mulching treatments in this experiment confirmed these results. The soil under mulching treatments ST and GT had significantly higher LOC, DOC, POC and EOC concentrations in the surface 0–40 cm layer than those with no mulching treatment (Table 2), probably attributable to the inputs of straw, root and its sections. Concentrations of labile C fractions in all treatments tended to decrease with soil depth (Table 2). However, the contents, distribution, and transfer of each labile C fraction differed from other studies. As discussed by us in Liu et al. [34], this could have been due to the fact that experiments were conducted on sloping land that had severe soil and water erosion, and therefore nutrient losses. Concentrations of total carbon and labile C fractions in all treatments showed seasonal dynamic change (Figs 3 and 4).

**Table 2:** Stocks of each SOC fraction and total SOC at depths of 0–40 cm and 40–100 cm.

Treatments	Soil depths	LOC kg m^–2^	DOC g m^–2^	POC kg m^–2^	EOC kg m^–2^	SOC kg m^–2^
CK	0–40 cm	0.15±0.01 b	0.89±0.04 b	0.80±0.04 b	0.65±0.05 b	3.19±0.10 c
	40–100 cm	0.05±0.00 a	0.76±0.02 b	0.31±0.02 b	0.31±0.04 b	1.63±0.10 a
	0–100 cm	0.21±0.01 b	1.65±0.03 b	1.11±0.03 c	0.97±0.05 b	4.82±0.19 c
ST	0–40 cm	0.17±0.02 a	1.10±0.05 a	1.18±0.06 a	0.79±0.05 a	3.52±0.12 b
	40–100 cm	0.06±0.00 a	0.84±0.03 a	0.42±0.02 a	0.39±0.05 b	1.72±0.07 a
	0–100 cm	0.23±0.01 a	1.95±0.04 a	1.60±0.04 a	1.18±0.05 a	5.24±0.18 b
GT	0–40 cm	0.18±0.01 a	1.08±0.06 a	1.08±0.07 a	0.78±0.06 a	3.80±0.16 a
	40–100 cm	0.06±0.00 a	0.87±0.04 a	0.38±0.03 a	0.48±0.04 a	1.85±0.11 a
	0–100 cm	0.24±0.01 a	1.95±0.05 a	1.46±0.05 b	1.26±0.05 a	5.65±0.16 a

**Fig 5:**
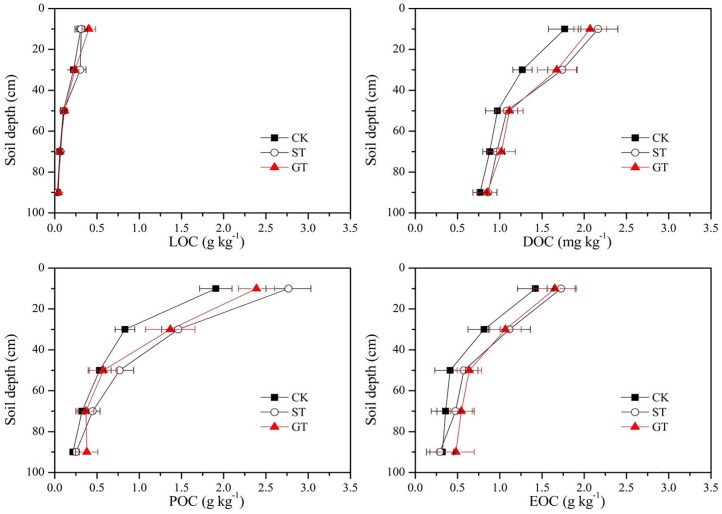
Content of Carbon fractions at different depths.

LOC is a short-term repository of soil nutrients and its main constituent is free state carbon [38]. It is characterized by rapid mineralization due to its labile nature, as it is not protected by soil colloids [39]. LOC can move with runoff and be easily lost, and has high decomposition and turnover rates [40, 1]. For these reasons LOC can be strongly affected by the seasons [41]. In the current experiment, LOC showed significant seasonal changes, where the maximum value occurred during February to March, after which content declined and remained lower prior to October (Fig 4). Lower accumulation of LOC during the period from April to September was possibly attributable to high decomposition of recent organic material inputs, and high loss with runoff at this rainy time [42]. Mulching practices did not alter the seasonal dynamic changes of LOC, but could increase its content, e.g., in March, ST and GT increased LOC by 167% and 122% respectively (Fig 4). In general, adoption of GT and ST increased LOC contents in the 0–100 cm soil profile by 0.102 g kg^–1^ and 0.136 g kg^–1^ respectively, compared to CK, and there was a 70–80% increase in the 0–40 cm layer (Fig 5). The higher values of LOC in ST and GT can possibly be attributed to the inputs from organic materials and root residues, as well as decreased losses with surface runoff as a result of mulching [43–44].

The DOC concentration is considerably lower than those of other labile C fractions, generally not more than 200 mg kg^–1^, but it is the most mobile fraction of SOC. It controls the turnover of nutrient and organic matter by affecting the development of microbial populations. In this experiment, ST and GT treatments significantly increased soil DOC concentrations at depths of 0–40 cm, by 28.56% and 23.33% respectively, (Fig 5) compared to CK, but there was no difference between ST and GT treatments at each layer of the soil profile. The increase in DOC with ST may be due to the soluble decomposed organic materials of the straw, while the increase in DOC with GT could possibly be attributed to an increase in organic acids and water-soluble carbohydrates from rhizodeposition and root exudates. In addition, a decrease in surface runoff under GT and ST was an important reason for the increased DOC, as DOC may be lost with runoff. Compared with CK, the DOC in GT and ST was favorably leached, deposited and absorbed into the subsoil layer, resulting in higher concentrations of DOC at depths of 20–40 cm (Fig 5). This was probably because of low soil bulk density in ST, and in GT lower pH would have increased DOC adsorption by soil [45]. However, there were no significant influences of GT and ST on DOC at depths of 40–100 cm.

The POC was more stable than other labile C fractions, because part of it can be combined with sand and thus avoid rapid decomposition. Generally, POC is composed of plant-derived remains as well as microbial and micro faunal debris [20], and hence soils under large annual return of litter are more conducive to the formation of POC [46]. In this study, a significant increase in POC was found in GT and ST treatments compared to CK, indicating that the residue of newly decomposed straw in ST, or return of aboveground residue and the rhizodeposition of the clover in GT, might enter the particulate fraction of POC. Input of organic debris promotes the development of stable micro-aggregates within macro-aggregates [47], and stable soil aggregates help to protect POC from rapid decomposition [46]. This might be another reason for the increase in POC with mulching treatments. The concentration of POC in the soil profile decreased with depth, and the increase of POC in GT and ST was mainly focused in the 0–40 cm soil layer (54.46% and 37.18% respectively). However there was no significant difference between GT and ST in POC concentration in each layer of soil profile (Fig 5). Similar results have been observed, e.g., Franzluebbers and Stuedemann [48], and Wander and Yang [44] found higher POC at the soil surface, and larger size POC at lower depth in a no-tillage with residue mulch experiment. Bastian et al. [31] reported that the formation of POC was often in relatively cold seasons, while the depletion of POC was in hot seasons, because the increase in soil temperature and moisture enhanced the rate of decomposition of POC [49]. But in fact, this rule is not absolute: if the rate of production of new POC was greater than the rate of POC decomposition, POC would show a net accumulation, even in the hot season. In this study, the high concentration of POC in GT and ST treatments occurred in July and September (Fig 4), probably due to the greater input of newly decomposed residue materials at this time.

EOC is characterized as being easily oxidizable due to the labile nature of its’ constituents [22], which include readily decomposed humic material and polysaccharides [50, 51]. Soil temperature and soil moisture have a substantial effect on EOC content [52]. Increased soil temperature and moisture during the period from June to September (Fig 4) may have been responsible for elevated EOC levels during this time (Fig 2). High temperatures would have enhanced the decomposition of new organic material and increased EOC by boosting microbial activity [41]. During July and August rain was torrential and the sandy loam soil of the experimental site was waterlogged. Increased moisture content in the soil (Fig 2) restricts the circulation of oxygen, leading to the creation of an anaerobic environment in the soil capillaries. Anaerobic conditions cause a reduction in the rate of decomposition of EOC [53]. Compared to the CK control, ST and GT soils had higher EOC content (Fig 4), indicating that mulching practices can significantly increase EOC content of the soil. A possible reason is that mulching contributes a further source of C to EOC. In addition, mulching may increase infiltration of water into the soil, resulting in high soil moisture content (Fig 2), effectively enhancing the anaerobic environment of the soil [30] and so further reducing EOC decomposition.

### The stocks of each SOC fraction

Sloping land is characterized by large amounts of runoff and low SOC storage [54–55]. This is typical of our experimental site in Danjangkou reservoir area [34]. The region experiences intense rainfalls from July to August, which, combined with the sandy loam soil, contributes to greatly increased soil moisture (Fig 2). We found that total SOC stock at depths of 0–100 cm in CK, ST and GT treatments was 4.82, 5.24 and 5.65 kg m^–2^, respectively (Table 2). These SOC values differ from those in other research on sloping land. The differences can be attributed to the variation in soil types in the other studies, as well as the use of different mulching materials and application rates. Some examples include 4.11 kg m^–2^ applied in a film mulching treatment, using plastic film to cover the soil surface [12], 3.96 kg m^–2^ in pebble mulching [13] and 5.48 kg m^–2^ in straw mulching treatments [56].

Compared with non-mulching treatments, mulching practices ST and GT increased total SOC stock by 10.34% and 19.12% respectively. Similar results were also reported in semi-arid shrub ecosystems in a developed country [57], and a developing country [11]. However, some reported no increase, or a decrease of total SOC stock under other mulching practices, e.g., film mulching [12] and pebble mulching [13]. The addition of organic materials into soil under ST and GT lead to significantly higher SOC stock than other cover materials [58]. The difference in SOC stock between GT and ST (Table 2) could have been due to the fact that the increase in soil organic carbon content in GT was relatively stable, and not as easily lost with runoff as it was in ST [34, 59].

The increases in labile soil organic carbon stock under GT and ST, when compared with the non-mulching control, were found mainly in the 0–40 cm layer. The LOC, DOC, POC, and EOC stocks for ST and GT show an increase of 0.02 and 0.03 kg m^–2^, 0.21 and 0.19 g m^–2^, 0.38 and 0.28 kg m^–2^, 0.14 and 0.13 kg m^–2^, respectively, compared with CK. The POC stock shows 35–48% increase over the 0–40 cm layer as a result of mulching, significantly higher than 13–20% increase for LOC, 21–24% increase for DOC, and 20–22% increase for EOC. This was consistent with other results [60], which indicated POC is more stable than other labile C fractions. Between GT and ST, there was no significant difference in the LOC, DOC, and EOC stocks in the upper 100 cm, unlike the POC stock.

### Relationships and proportion of SOC fractions

The Pearson correlation coefficients between the soil moisture (SM), soil temperature (SH) and the soil carbon fractions at depths of 0–20 cm were analyzed (Table 3).

**Table 3:** Pearson correlations among soil moisture (SM), soil temperature (SH) and SOC fractions at depth 0–20 cm.

	SH	SM	SOC	LOC	DOC	POC	EOC
SH	1						
SM	0.890**	1					
SOC	-0.549**	-0.330	1				
LOC	-0.175	0.057	0.505*	1			
DOC	-0.443*	-0.209	0.568**	0.210	1		
POC	0.561**	0.594**	0.088	0.044	0.183	1	
EOC	0.557**	0.636**	0.032	0.010	0.231	0.601**	1

**. Correlation is significant at the 0.01 level (2-tailed),

*. Correlation is significant at the 0.05 level (2-tailed).

In general, SOC and DOC negatively correlated with soil water content, and more particularly soil temperature, which had significantly linear correlations. This suggests that soil temperature might be a major cause for diminished soil SOC and DOC. In this study, the use of ST and GT decreased soil temperature compared to CK, which would account for the increase in SOC and DOC in these treatments. POC and EOC had positive correlations with soil temperature and with soil water content, however the correlation coefficients of POC and EOC with soil temperature (r = 0.561 and 0.557, respectively) were lower than those with soil water content (r = 0.594 and 0.636, respectively). This indicated that soil water content and soil temperature jointly affected the formation or decomposition of POC and EOC, and the change of soil water content might be more important than soil temperature in affecting POC and EOC.

Many previous studies indicated that there were significant and positive correlations between LOC, DOC, POC, EOC and SOC [60–62], and they suggested that LOC, DOC, POC and EOC were suitable indicators for evaluating soil quality and C changes in management practices. However, in this study, only LOC and DOC significantly and positively correlated with SOC, which suggested that LOC and DOC were more sensitive than other labile C fractions. Wan et al. [63] also reported that LOC and DOC were the primary energy source for soil microorganisms, and control both the nutrient turnover and the development of microbial populations that play an important role in soil chemical and biological processes. By incubating bulk soil and density fractions, former researchers observed that LOC was the driving factor in soil respiration [64–65], due to its high C concentration.

The changes in soil organic carbon contents in relation to soil temperature and soil moisture indicate that seasonal temperature and rainfall patterns of a particular region will affect carbon sequestration. Our study showed that mulching with both ST and GT might have beneficial effects on sloping land. According to the seasonal patterns in our study area, we suggest that the optimal period for straw mulching is between December and early the following June. For the planting of live grass cover on sloping land, we suggest that the optimal period is between March and May. This would ensure that the grass would be well established from June to September, providing protection from the loss of soil and water during the rainy season.

## Conclusions

A four-year field experiment demonstrated that the adoption of GT and ST could significantly increase the contents of total SOC and SOC fractions in sloping arable land. Compared with CK, the stocks of total SOC, LOC, DOC, POC, and EOC at depths of 0–100 cm increased significantly in the mulching treatments. GT had greater concentrations and stocks of SOC and it’s fractions than ST. Our study confirms that mulching measures, especially GT mulching, are promising management options for enhancing soil C sequestration in sloping arable land in the Xiaofuling watershed of Danjiangkou reservoir area of China.

Our study is the first investigation of the dynamic changes of SOC and its active fractions in sloping land under soil surface mulching. Results showed that SOC, DOC and LOC accumulated in cold and dry seasons, while POC and EOC accumulated in warm and humid seasons.

## Supporting Information

S1 File Highlights. (DOCX)

## Abbreviations

DOC: Dissolved organic carbon
EOC: Easily oxidizable organic carbon
LOC: Light fraction organic carbon
LOM: Light fraction organic matter
POC: Particulate organic carbon
POM: Particulate organic matter
SOC: Soil organic carbon
