# Correction: Dynamic Changes of Soil Surface Organic Carbon under Different Mulching Practices in Citrus Orchards on Sloping Land

**DOI:** 10.1371/journal.pone.0175600

**Published:** 2017-04-06

**Authors:** Chiming Gu, Yi Liu, Ibrahim Mohamed, Runhua Zhang, Xiao Wang, Xinxin Nie, Min Jiang, Margot Brooks, Fang Chen, Zhiguo Li

An affiliation for the 3rd author is not indicated. Ibrahim Mohamed is also affiliated with: Soil and Water Department, Faculty of Agriculture, Benha University, Egypt.
