# Supplementary material for: Dynamic Changes of Soil Surface Organic Carbon under Different Mulching Practices in Citrus Orchards on Sloping Land
Source: PLoS One. 2016 Dec 28;11(12):e0168384. doi: 10.1371/journal.pone.0168384 (PMC5193398; doi:10.1371/journal.pone.0168384)
Supplement: S1 File — (DOCX) [file pone.0168384.s001.docx]

**Highlights**

- We assess the influence of agricultural management change on carbon sequestration.
- A field experiment of carbon cycle in a cotton field was conducted over two years.
- We measured the annual carbon balance in different management strategies
- Mulching cultivation is an effective way to increase carbon sequestration in arid areas.
